# Unraveling the Impact of Aging on the Structural, Magnetic, and Superconducting Properties of 2G HTS Tapes

**DOI:** 10.3390/ma19122486

**Published:** 2026-06-10

**Authors:** Paweł Pęczkowski, Jarosław Piętosa, Piotr Zachariasz, Paweł Gąsior, Ryszard Zalecki, Jan Marek Michalik, Łukasz Gondek, Anna Krztoń-Maziopa

**Affiliations:** 1 Institute of Physical Sciences, Faculty of Mathematics and Natural Sciences, School of Exact Sciences, Cardinal Stefan Wyszyński University, K. Wóycickiego 1/3 Street, 01-938 Warsaw, Poland; 2 Group of Phase Transition, Division of Physics of Magnetism, Institute of Physics, Polish Academy of Science, Lotników 32/46 Avenue, 02-668 Warsaw, Poland; pietosa@ifpan.edu.pl; 3 Center for Functional Materials, Łukasiewicz Research Network—Institute of Microelectronics and Photonics, Zabłocie 39 Street, 30-701 Kraków, Poland; piotr.zachariasz@imif.lukasiewicz.gov.pl; 4 Laboratory of Laser Plasma Spectroscopy, Institute of Plasma Physics and Laser Microfusion (IPPLM), Hery 23 Street, 01-497 Warsaw, Poland; pawel.gasior@ifpilm.pl; 5 Department of Solid State Physics, Faculty of Physics and Applied Computer Science, AGH University of Kraków, Mickiewicza 30 Avenue, 30-059 Kraków, Poland; zalecki@agh.edu.pl (R.Z.); jmichali@agh.edu.pl (J.M.M.); lgondek@agh.edu.pl (Ł.G.); 6 Faculty of Chemistry, Warsaw University of Technology, Noakowskiego 3, 00-664 Warsaw, Poland; anna.maziopa@pw.edu.pl

**Keywords:** GdBCO superconductor, 2G HTS tape aging, critical current density, structural analysis, SQUID magnetometry, LIBS technique

## Abstract

Second-generation high-temperature superconducting tapes (2G HTS; SuperPower Inc., Glenville, NY, USA) based on GdBCO (GdBa_2_Cu_3_O_7−*δ*_, where *δ* denotes oxygen deficiency) were aged at −26.4 °C, +2 °C, and room temperature (RT) to evaluate the degradation of their superconducting properties. HTS tapes stored at RT exhibited a significantly higher deterioration rate compared to those maintained at lower temperatures. Laser-induced breakdown spectroscopy (LIBS) analysis demonstrated a gradual reduction in the effective chemical depth-profiling length over time, indicating a correlation between the degradation mechanism and the reduction in the effective volumetric density of the GdBCO superconducting layer. These findings imply that oxygen diffusion or redistribution processes substantially contribute to the long-term degradation of GdBCO-based HTS tapes.

## 1. Introduction

Recent years have witnessed considerable progress in both laboratory research [1,2,3] and large-scale manufacturing of superconducting tapes [4,5]. Among various alternatives, Gd- or Y-based superconducting tapes have been identified as particularly appropriate for fabricating devices capable of handling high currents and voltages [6,7,8]. 2G HTS tapes are produced by several manufacturers, including SuperPower Inc. (Glenville, NY, USA) [9,10], a company within the Furukawa Electric Group. SF (stabilizer-free) tapes are specifically designed for use in AC (alternating current) devices. They have a substrate layer made of a non-magnetic material with high resistivity, resulting in low losses during the flow of AC.

Deterioration of the silver (Ag) protective layer can significantly impact the tape’s performance. Moreover, 2G HTS tapes have considerable potential with the space industry [3], as they may provide protection for spacecraft and crew against cosmic radiation and solar wind [11,12]. Cosmic radiation exposure remains a major challenge for long-duration space missions, including expeditions to Mars [13]. Consequently, the degradation of superconducting tapes becomes a significant concern. The assembly of protective coatings presumably explains the sporadic reports of corrosion or aging phenomena within the GdBCO layer. Typical protective coatings are usually composed of silver (Ag) [14], whereas less common alternatives include gold (Au) [15], adhesive Kapton [16], or strontium titanate (SrTiO_3_) [17].

Several reports concerning GdBCO films have been published by Li et al. [18] and Schlesier et al. [19], describing changes in various superconducting parameters over time. Investigation of individual GdBCO layers and GdBCO-based tapes shielded with Au protective coating demonstrated significant deterioration in superconducting properties, including the critical temperature (*T*_c_) and critical current density (*J*_c_), under ambient conditions.

The neutron diffraction pattern of YBa_2_Cu_3_O_7−*δ*_ (YBCO) maintained in vacuum over four years shows no evidence of structural distortion or phase separation attributable to aging [20]. Electrical resistivity measurements also do not indicate a significant modification in the critical temperature (*T*_c_) values. Furthermore, it is well established that the superconductivity of *RE*Ba_2_Cu_3_O_7−*δ*_ (*RE*—rare-earth element) type materials is sensitive to the oxygen content within the unit cell and its local symmetry [21]. However, the critical temperature (*T*_c_ ≈ 90 K) remains largely unaffected even when *δ* > 0.2 [21,22]. Nonetheless, concerns regarding the long-term performance of 2G HTS tape persist, potentially owing to alterations in oxygen arrangement [23], which may evolve and lead to gradual modifications of superconducting properties, thereby rendering long-term stability a pivotal consideration for practical applications [24]. Recent studies have shown that lattice strain, grain boundary effects, and inhomogeneity in perovskite materials such as LaMnO_3_ provide a framework for various material systems, enabling a better understanding of oxygen deficiency and microstrain evolution in the GdBCO structure [25,26].

This study investigated the aging of 2G HTS SF 12050 tapes manufactured by SuperPower Inc. (Glenville, NY, USA) based on GdBCO with an Ag protective layer. The study lasted 1, 3, and 5 years and was conducted under various temperature conditions, including −26.4 °C, +2 °C, and RT. The primary objective is to assess the long-term stability of their superconducting properties and to improve the understanding of the underlying deterioration mechanisms.

## 2. Materials and Methods

**Figure 1:**
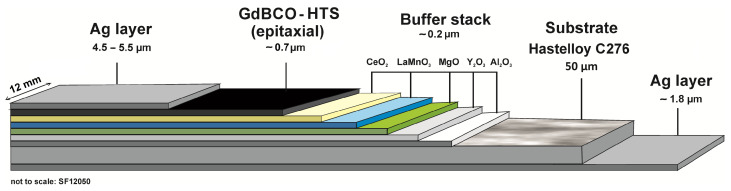
Cross-section of the 2G HTS SF (stabilizer-free) 12050 superconducting tape by SuperPower Inc. (Glenville, NY, USA) [9,10].

The commercially available second-generation high-temperature superconducting 2G HTS SF 12050 tape with an overall thickness of approximately 60 μm incorporates an embedded GdBa_2_Cu_3_O_7−*δ*_ (GdBCO) superconducting layer of ~0.7 μm thick. A schematic diagram of 2G HTS tape produced by SuperPower Inc. (Glenville, NY, USA) [9,10] is illustrated in Figure 1. This tape demonstrates a self-field critical current of 317 A per 12 mm width at 77 K [27].

SuperPower Inc.’s (Glenville, NY, USA) stabilizer-free superconducting tapes are manufactured using thin-layer technology [9,10]. A non-magnetic Hastelloy C276 alloy (Ni: 57.0%, Mo: 16.0%, Cr: 15.5%, Fe: 5.5%, W: 4.0%, Co: 2.5%) functions as the 50 μm thick substrate for the SF 12050 tape. During the fabrication process of 2G HTS tapes, a buffer stack is deposited onto a metal substrate, followed by an epitaxial GdBa_2_Cu_3_O_7−*δ*_ (GdBCO) layer. This layer attains superconductivity after annealing in an oxygen atmosphere and cooling below 90 K. Subsequently, a protective silver (Ag) coating, approximately 4.5 to 5.5 µm thick, is applied over the GdBCO layer (~0.7 µm), and the entire assembly is then laminated.

Pieces of the 2G HTS tape, each measuring 5 cm, were aged under various conditions, including different temperatures and aging durations. These samples were stored in chambers with controlled humidity at −26.4 °C, +2 °C, and RT, and their superconducting properties were monitored after 1, 3, and 5 years.

Scanning electron microscopy (SEM) was performed using a JEOL JSM-5900LV microscope (JEOL Ltd., Tokyo, Japan), equipped with a tungsten cathode electron gun. SEM images were collected in high-voltage (HV) mode with magnifications ranging from 2000× to 16,000×. Before SEM examination and X-ray diffraction (XRD) analysis, the silver (Ag) top coating on the 2G HTS tape (Figure 1) was carefully removed through mechanical polishing with commercially available 3.0 µm Luxor^®^ (Luxor^®^ Polishing Compounds, Belleville-en-Beaujolais, France) polishing paste, and final polishing was performed with 0.3 µm Luxor^®^ polishing paste. Subsequently, the exposed GdBCO layer was cleaned using isopropyl alcohol in an ultrasonic cleaner.

XRD patterns were acquired at ambient temperature utilizing a PANalytical Empyrean powder diffractometer with *K*_α_ radiation (*λ*_Cu_ = 1.54056 Å). The instrument was operated at 40 kV and 40 mA in the Bragg–Brentano geometry. Bragg reflection positions were calibrated using LaB_6_ (NIST660) as the reference standard. XRD patterns were recorded over the 2*θ* range of 4–100° with a step size of 0.02°. Since 2G HTS tapes are *c*-axis-oriented GdBCO systems [28,29], the Rietveld refinement was performed by summing only the in-plane texture. The resulting XRD data were analyzed employing the FullProf Suite software to determine structural and microstructural parameters [30].

The direct current (DC) magnetization of the aged 2G HTS tapes (0.28 × 0.28 cm^2^) was measured using a Quantum Design (Quantum Design GmbH, Pfungstadt, Germany), SQUID magnetometer (MPMS-5) within a temperature range of 5–120 K. A magnetic field of up to 50 kOe (~4000 kA/m) was applied parallel to the *c*-axis of the GdBCO structure. Following the application of demagnetization corrections [31], critical current densities (*J*_c_) and magnetization curves were recorded as functions of the internal magnetic field (*H*_int_).

Laser-induced breakdown spectroscopy (LIBS) [32] was employed to analyze the chemical composition of 2G HTS tapes. Plasma was generated using an Nd:YAG laser, focused through a plano-convex lens, delivering 0.5 mm diameter pulses with a duration of 8–12 ns and an energy of 50 mJ. A medium-resolution Mechelle spectrometer, operating with a 200 ns pulse delay and a 2 μs acquisition time, was utilized to identify the spectral lines. A low-resolution IBSEN (IBSEN Photonics, Farum, Denmark) spectrometer was used, providing satisfactory repeatability and a high signal-to-noise ratio (SNR) for quantifying chemical depth profiles. LIBS data were background-corrected using the rubberband algorithm (RBA) and vector normalization, with the initial pulse regarded as the cleaning pulse [33].

## 3. Results

### 3.1. Microstructural and Structural Analyses

**Figure 2:**
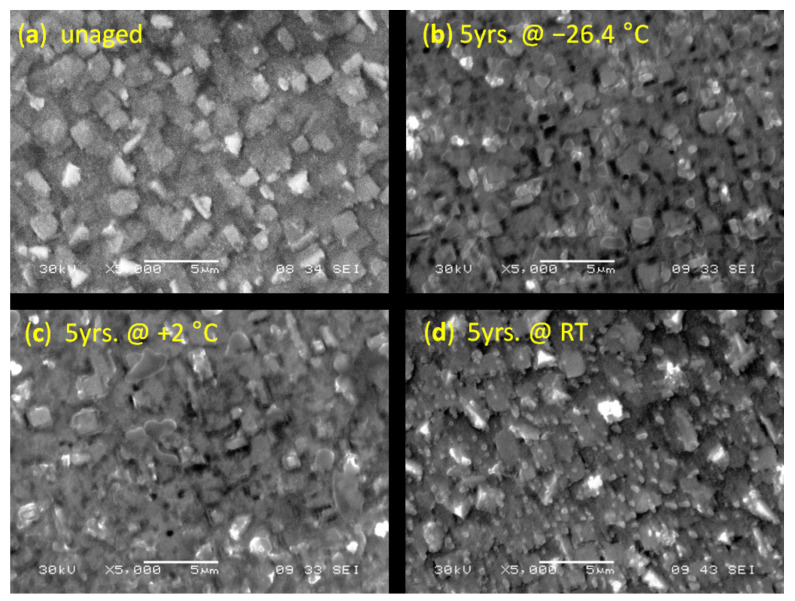
SEM images showing a characteristic microstructure of 2G HTS tape before aging (**a**) and after 5 years of storage at (**b**) −26.4 °C, (**c**) +2 °C, and (**d**) RT (atmospheric conditions).

The local microstructures of superconducting GdBCO layers, after the removal of the Ag layer, are depicted in Figure 2. All samples exhibit irregular GdBCO blocks of varying sizes. Small white spots observed in SEM images indicate residual Ag that was not fully removed during ultrasonic cleaning.

For the tape maintained at +2 °C, a few pits are observable (Figure 2c), which may be attributed to the porous buffer layer (Figure 1), suggesting that the GdBCO layer demonstrated reduced mechanical resistance to ultrasonic cleaning in this region.

**Figure 3:**
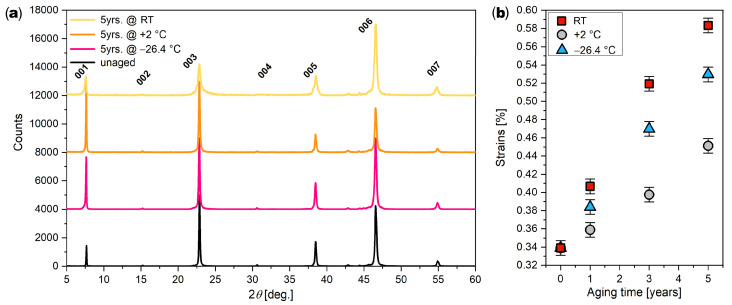
(**a**) XRD patterns of unaged 2G HTS tape and tapes conditioned for 5 years with (**b**) microstrains derived for all investigated materials.

X-ray diffraction (XRD) patterns reveal almost ideal texture along the *c*-axis perpendicular to the GdBCO blocks. As illustrated in Figure 3, solely (00*l*)-type Bragg reflections are identified in the analyzed materials. Therefore, a Rietveld refinement was restricted to the *c*-lattice parameter and strains contributing to this crystallographic direction. Due to such a well-textured GdBCO film, no insight into the orthorhombicity of the crystal structure can be provided. Minor diffraction lines within the 2*θ* range of 40–45° may originate from residual silver (Ag), Hastelloy, or buffer layers, all components of 2G HTS tape. For the specimen stored for 5 years under ambient conditions, the Bragg reflections are significantly broader compared to those observed in other 2G HTS tapes.

Given that the microstructure of the samples did not exhibit significant changes, the observed broadening of the diffraction reflections is presumably attributable to the development of lattice strains. Such strains are generally associated with the formation of structural defects, including dislocations, vacancies, and other crystallographic imperfections. For the studied GdBCO tapes, a monotonic (quasi-linear) increase in strain was observed, with the slope varying with storage conditions (Figure 3). It is noteworthy that after 5 years of aging under ambient conditions, the additional microstrain induced in the 2G HTS tape was approximately 27.5% greater than that in the specimen stored at 26.4 °C. The observed evolution of microstrain correlates with the degradation of superconducting performance in the 2G HTS tape, presumably attributable to an increasing concentration of oxygen vacancies within the GdBCO structure.

### 3.2. Magnetic and Superconducting Properties

**Figure 4:**
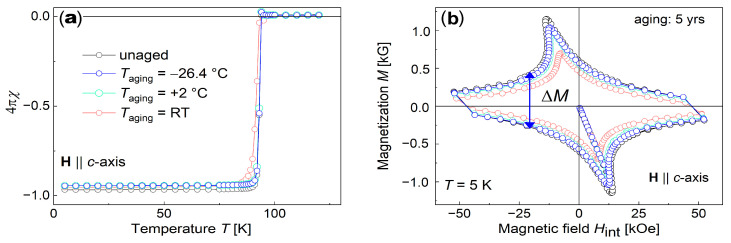
(**a**) The magnetic susceptibility and (**b**) magnetization curves recorded at *H*_ext_ = 10 Oe parallel to the *c*-axis, for 2G HTS tapes aged at different conditions for 5 years.

A strong diamagnetic response endures up to a critical temperature (*T*_c_) of 93 K across all 2G HTS tapes (Figure 4a), indicating that superconductivity is maintained for 5 years. Nevertheless, a noticeable decline in superconducting characteristics has been observed over time, depending on the conditions under which the samples were preserved. This deterioration is evidenced by a marginal weakening of diamagnetism and a decrease in *T*_c_.

Figure 4b illustrates hysteresis loops of GdBCO layers in an internal magnetic field aligned parallel to the *c*-axis at a temperature of 5 K. Based on these magnetization curves, the critical current density (*J*_c_) was precisely estimated utilizing the proportionality of Δ*M* (the difference between ascending and descending magnetic field cycles), as derived from Bean’s critical state model [34]:

(1) $$J_{c}=\frac{20\cdot \Delta M}{a\left(1-\frac{a}{3b}\right)}$$

where *J*_c_ is expressed in A·cm^−2^, Δ*M* is measured in G (Gauss), and *a* and *b* are the sample dimensions (in cm) in the plane perpendicular to the applied magnetic field (*H*_ext_ = 10 Oe).

**Figure 5:**
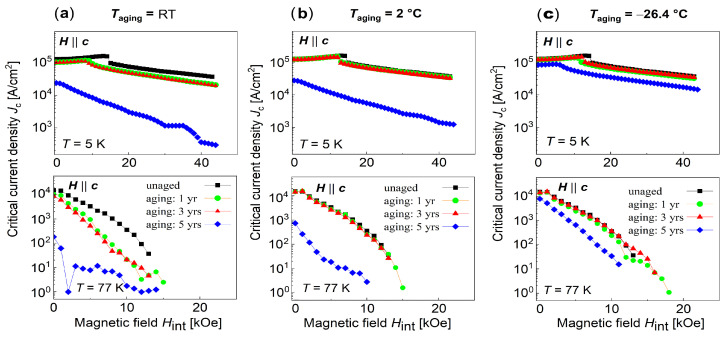
Critical current densities (*J*_c_) of 2G HTS tapes stored (**a**) at RT (atmospheric conditions), (**b**) at +2 °C, and (**c**) at −26.4 °C, determined at low temperatures: 5 K (**upper**) and 77 K (**lower**) at a magnetic field parallel to the *c*-axis.

The critical current densities (*J*_c_), evaluated in accordance with Equation (1), are illustrated in Figure 5. The upper panels depict the magnetic data at 5 K, whereas the lower panels display magnetic curves at 77 K. For all evaluated 2G HTS tapes, a decrease in *J*_c_ of at least one order of magnitude is observed after 5 years of aging. The most significant deterioration of superconductivity is evident for tape maintained under ambient conditions. Except for the sample stored at −26.4 °C, the critical currents exhibit similar characteristics both at 5 K and 77 K. Additionally, the superconducting properties at 77 K of the tape stored at RT exhibited substantial deterioration after only 1 year of aging. Conversely, the superconducting properties of the 2G HTS tape stored at the lowest temperature for the same period remained stable.

### 3.3. Laser-Inducted Breakdown Spectroscopy (LIBS)

Laser-induced breakdown spectroscopy (LIBS) is a non-contact method for analyzing the chemical composition of materials as a function of depth. It offers notable advantages for analyzing multilayer materials, coatings, or structures with compositional gradients, as well as for evaluating deterioration levels and surface contamination. The LIBS technique represents a variant of atomic emission spectroscopy that utilizes a high-intensity laser pulse to generate a microscopic plasma discharge on the sample surface. Currently, the LIBS method is employed across numerous industrial [35], environmental [36], and scientific [37] fields, including extraterrestrial explorations on Mars [38].

This investigation examines the evolution of chemical depth profiles within the GdBCO layer throughout aging. LIBS measurements were prepared in a vacuum of 5 × 10^−5^ mbar to prevent interference from atmospheric oxygen. Standard experimental configurations, as referenced in the literature [39], were utilized primarily due to limited sample size, rather than modifications to optimize laser exposure and spectroscopic observation conditions, which are typically necessary for such analyses.

**Figure 6:**
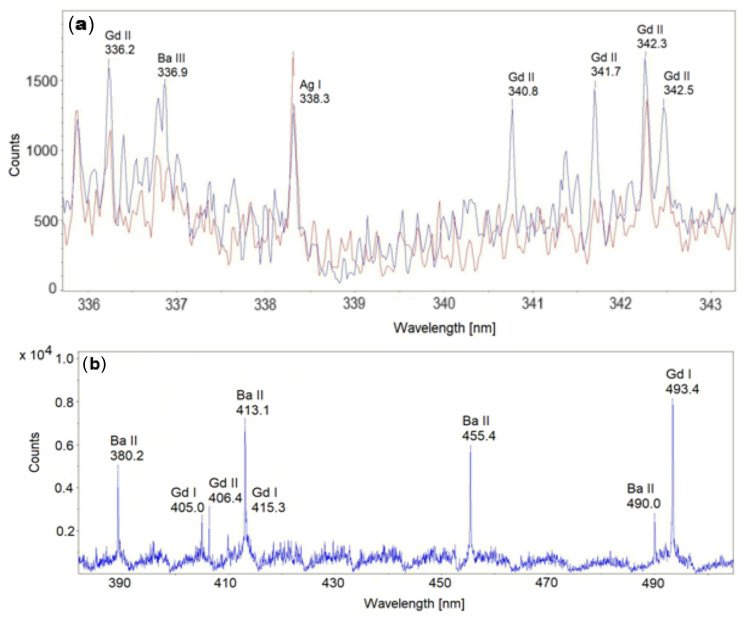
Sample LIBS results of 2G HTS tape stored at −26.4 °C over 3years, including (**a**) spectra after the 1st laser pulse (in orange, Ag-dominated spectrum) and after the 2^nd^ laser pulse (in blue, Ba- and Gd-dominated spectrum), (**b**) the spectral region with characteristic lines of Ba and Gd after the 3^rd^ laser pulse.

Figure 6 presents initial LIBS spectra, which identify the Gd and Ba elements, constituents of the superconducting layer in 2G HTS tape. The Ag line (338.3 nm) diminishes rapidly after the initial pulse, whereas the combined peaks corresponding to Ba at 455.4 nm and Gd at 493.4 nm become more prominent following subsequent laser pulses (II and III). It indicates that the GdBCO layer retains a small amount of residual silver (Ag) that did not evaporate during the first laser ablation. As the laser pulses continue, the chemical profile primarily reflects the characteristic line of Gd and Ba, consistent with the composition of the GdBCO layer.

**Figure 7:**
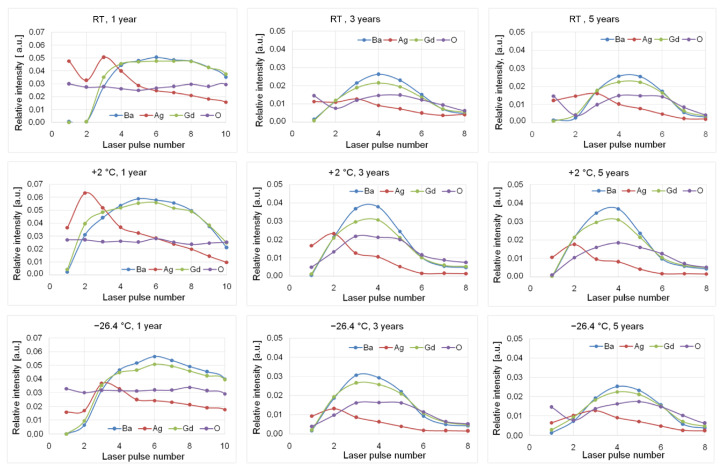
Chemical depth profiles of HTS tapes stored at RT (atmospheric conditions), +2 °C, and −26.4 °C for 1, 3, and 5 years.

Chemical depth profiling of the GdBCO layers (Figure 7) revealed differences among 2G HTS tapes aged under various conditions. For samples aged for 1 year, nearly identical profiles were observed, indicating that this duration does not substantially influence the chemical composition. Conversely, at prolonged aging periods, the oxygen profiles gradually diminish, indicating depletion of oxygen within the superconducting layers.

## 4. Discussion and Conclusions

This research elucidates that thermodynamic conditions, particularly ambient-temperature humidity, profoundly influence the superconductivity of 2G HTS tapes. Structural, microstructural, and magnetometry results suggest that atmospheric moisture is the principal factor contributing to the deterioration of superconductivity in 2G HTS tapes. Microstructural analysis corroborates this hypothesis, exhibiting consistent degradation XRD patterns across all samples and excluding mechanical macro-cracking as the primary degradation mechanism under these specific aging conditions.

The observed broadening of XRD peaks suggests the development of microstrains due to structural disorder or a lattice mismatch between the buffer stack and the GdBCO superconducting layer. Fluctuations in oxygen stoichiometry likely drive this phenomenon. Therefore, combined XRD and LIBS data indicate that the oxygen content in the epitaxial GdBCO layers (~0.7 µm) decreases significantly during aging. This oxygen loss is the primary driving force behind the suppression of superconductivity in 2G HTS tapes and the weakening of flux pinning centers.

Aging-related degradation of the GdBCO layer is a complex interfacial process governed by interactions between the silver coating and the Hastelloy substrate and driven by oxygen diffusion across these interfaces. Nevertheless, the primary reason for the superconductivity deterioration remains the localized deoxygenation of the GdBCO lattice, which induces the formation of strains, finally leading to suppression of superconductivity. Experimental findings underscore a significant disparity in durability across various storage conditions: ambient aging produces the most pronounced tape degradation. After a period of 5 years, the critical current density (*J*_c_) at 77 K diminished by approximately two orders of magnitude. Conversely, samples maintained at cryogenic temperatures (−26.4 °C) under stable, low-humidity environments demonstrated negligible degradation, thereby preserving superconducting parameters comparable to those of pristine 2G HTS tapes.

## Data Availability

The raw data supporting the conclusions of this article will be made available by the authors on request.
